# Reduced tear fluid production in neurological diseases: a cohort study in 708 patients

**DOI:** 10.1007/s00415-023-12104-3

**Published:** 2023-12-08

**Authors:** Elena Luib, Antonia F. Demleitner, Isabell Cordts, Erica Westenberg, Petra Rau, Dominik Pürner, Bernhard Haller, Paul Lingor

**Affiliations:** 1 Department of Neurology, Klinikum rechts der Isar, School of Medicine, Technical University of Munich, Ismaninger Str. 22, 81675 Munich, Germany; 2 Institute of AI and Informatics in Medicine, Klinikum rechts der Isar, Technical University of Munich, Munich, Germany; 3 DZNE, German Center for Neurodegenerative Diseases, Munich, Germany; 4 Munich Cluster for Systems Neurology (SyNergy), Munich, Germany

**Keywords:** Tear fluid, Wetting length, Neurological diseases, Neurodegeneration

## Abstract

**Background:**

Tear fluid (TF) production is an important component of normal ocular function. It is regulated by parasympathetic and sympathetic innervation. Because parasympathetic nerve fibers originate in the brainstem, pathology in this brain region may affect TF production. For example, a reduction in TF production has been described in patients with Parkinson’s disease (PD).

**Methods:**

TF was collected at one center from 772 individuals, 708 of which were patients with different neurological diseases, and 64 healthy controls. Wetting lengths (WL) were recorded using Schirmer test strips with a collection time of 10 min.

**Results:**

WL correlated negatively with age and was significantly reduced in subgroups of patients with neurodegenerative diseases (NDDs) (PD, Amyotrophic lateral sclerosis (ALS), other motor neuron diseases (MNDs)), as well as inflammatory/autoimmune/infectious central nervous system (CNS) diseases and vascular CNS diseases (VCDs), even if corrected for age or sex. While temperature had a significant negative effect on TF production, other environmental factors, such as hours of sunlight and humidity, did not.

**Conclusion:**

WL was altered in many neurological diseases compared to healthy controls. Most importantly, we observed a reduction of WL in NDDs, independent of age or sex. This study highlights the potential of WL as an easily obtainable parameter and suggests functional alterations in the autonomic innervation in various neurological disorders.

**Supplementary Information:**

The online version contains supplementary material available at 10.1007/s00415-023-12104-3.

## Introduction

Tear fluid (TF) is an indispensable component of physiological ocular function. Sufficient production is needed for cleansing, lubricating and refractory purposes. Dry eye syndrome, marked by significantly reduced production, is a frequent complaint among middle- to older-aged adults and can result in damage to the ocular surface [39]. The lacrimal glands receive both parasympathetic and sympathetic innervation [14]. While the parasympathetic nervous system, whose preganglionic fibers originate in the brainstem (pontine tegmentum, superior salivatory nucleus), stimulates TF secretion, the sympathetic nervous system (originating in the spinal cord) inhibits TF secretion, presumably by causing vasoconstriction [31].

Analysis of TF as potential biomarker fluid is of particular interest as the eye is developmentally an externally accessible extension of the brain. Because of the involvement of the brainstem in the parasympathetic innervation of the lacrimal glands, an alteration in lacrimal secretion is an easily detectable sign of autonomic dysfunction in neurological disorders (NDs) and could contribute to early diagnosis. Many brain pathologies have ocular manifestations, for example stroke, multiple sclerosis (MS), Parkinson’s disease (PD) and Alzheimer's disease [28]. Previous studies have shown that proteins and miRNAs can be extracted from TF and used as biomarkers [23]. Importantly, recent studies have shown that the detection of biomarkers in tear fluid, for example misfolded prion protein, is more sensitive than that in blood [38].

However, there have been few studies addressing TF production in neurological patients, and these have mostly studied small patient cohorts and focused on particular disorders [20]. Significantly reduced TF wetting lengths (WL) using the Schirmer tear test (STT) have been demonstrated in patients with PD in multiple studies [4, 6, 24] and alterations in TF production have been also reported in other NDs [12, 21, 22, 25].

In this study, we quantified TF volume by measuring the WL with the STT in a comprehensive cohort of 708 patients with different NDs. We demonstrate significantly decreased TF volumes in several disease groups.

## Materials and methods

### Study design and participants

This prospective, monocentric study was conducted between September 2019 and December 2021 at the Department of Neurology of the University Hospital rechts der Isar in Munich, Germany. TF was collected from 708 consecutive patients with NDs in outpatient clinics and neurological inpatient wards, as well as from 64 healthy volunteers without any signs of NDs. The detailed characteristics of the cohort are given in Table 1. Patients were divided into 9 subgroups according to the primary reason for their hospitalization or visit: Amyotrophic lateral sclerosis (ALS), other motor neuron diseases (MNDs), PD, other neurodegenerative disease (NDDs), neuromuscular diseases, tremor syndromes, inflammatory/ autoimmune/ infectious central nervous system (CNS) diseases, stroke and vascular CNS diseases (VCDs), and other NDs. ALS patients were included if the diagnosis was probable according to the revised El Escorial criteria. PD patients were included if PD was a probable diagnosis according to the Movement Disorder Society clinical diagnostic criteria. No other inclusion or exclusion criteria regarding age, sex, disease duration or therapeutic intervention were applied. Written informed consent was obtained from all participants. The study complies with the Declaration of Helsinki and was approved by the Ethics Committee of the Technical University of Munich, School of Medicine (approval number: 9/15S).

**Table 1:** Demographic and clinical data of the ND subgroups

	ALS	Other MND	PD	Other NDD	NMD	Tremor	Inflamm. CNS diseases	VCD	Other ND	Control group
N	65	29	94	36	22	10	87	135	230	64
Sex										
Male										
N (%)	38 (58.46)	17 (58.62)	58 (61.70)	21 (58.33)	12 (54.55)	4 (40.00)	34 (39.08)	51 (37.78)	95 (41.30)	26 (40.63)
Female										
N (%)	27 (41.54)	12 (41.38)	36 (38.30)	15 (41.67)	10 (45.45)	6 (60.00)	53 (60.92)	84 (62.22)	135 (58.70)	38 (59.38)
Age										
Mean ± sd	65.26 ± 12.72	37.52 ± 16.9	68.10 ± 10.53	69.31 ± 12.89	56.64 ± 18.67	60.60 ± 20.92	42.92 ± 15.11	69.96 ± 14.80	54.57 ± 19.83	45.30 ± 21.06
Median (min–max)	66 (36–88)	35 (17–82)	70 (37–85)	71.50 (27–94)	57.50 (25–82)	67 (18–84)	40 (18–77)	74 (24–97)	55 (17–90)	36 (22–88)
Clinical data										
Disease duration (years)										
Mean ± sd	2.74 ± 4.54	18.84 ± 14.44	6.48 ± 5.63	2.62 ± 2.19	8.77 ± 14.99	7.58 ± 9.54	4.57 ± 8.30	0.42 ± 1.73	2.17 ± 6.12	NA
Median (min–max)	1.58 (0.25–33.58)	16.58 (1.67–46.67)	5.08 (0–25)	2.83 (0–6.83)	2.67 (0.08–49.75)	3.42 (0.33–29.92)	0.42 (0–49.83)	0 (0–14.50)	0 (0–50)	NA
Ophthalmological data wetting length (mm/10 min)										
Mean ± sd	11.70 ± 10.42	19.65 ± 10.32	10.82 ± 7.78	10.67 ± 8.32	17.34 ± 12.95	15.06 ± 12.75	14.59 ± 10.58	10.84 ± 8.85	13.53 ± 10.51	23.79 ± 11.42
Median (min–max)	8 (1–35)	17.25 (4–35)	9.50 (1–35)	8.50 (1–31)	13.50 (1.5–35)	9.50 (2–35)	10.50 (1–35)	8 (1–35)	10.50 (1–35)	27 (1.5- 35)
95% CI	9.05–14.35	15.65–23.65	9.19–12.45	7.81–13.53	11.60–23.08	5.25–24.86	12.33–16.84	9.31–12.37	12.14–14.91	20.91–26.66
Ophthalmic disease^A^										
Cataract										
N (%)	4 (6.15)	1 (3.45)	21 (22.34)	3 (8.33)	2 (9.09)	1 (10.00)	1 (1.15)	32 (23.70)	21 (9.13)	5 (7.81)
Glaucoma										
N (%)	3 (4.62)	0 (0)	6 (6.38)	0 (0)	1 (4.55)	0 (0)	1 (1.15)	7 (5.19)	4 (1.74)	0 (0)
Inflammatory										
N (%)	0 (0)	0 (0)	3 (3.19)	3 (8.33)	1 (4.55)	0 (0)	2 (2.30)	5 (3.70)	9 (3.91)	0 (0)
Autoimmune										
N (%)	0 (0)	0 (0)	0 (0)	0 (0)	0 (0)	0 (0)	0 (0)	0 (0)	1 (0.43)	0 (0)
None										
N (%)	57 (87.69)	28 (96.55)	65 (69.15)	27 (75.00)	18 (81.82)	8 (80.00)	76 (87.36)	90 (66.67)	183 (79.57)	58 (90.63)
Contact lenses										
Yes										
N (%)	2 (3.08)	1 (3.45)	1 (1.06)	2 (5.56)	0 (0)	0 (0)	5 (5.75)	0 (0)	12 (5.22)	7 (10.94)
No										
N (%)	63 (96.92)	28 (96.55)	93 (98.94)	34 (94.44)	22 (100.00)	10 (100.00)	82 (94.25)	135 (100.00)	218 (94.78)	57 (89.06)
Eye medication^A^										
Prostaglandin receptor agonists										
N (%)	1 (1.55)	0 (0)	4 (4.26)	0 (0)	1 (4.55)	0 (0)	0 (0)	5 (3.70)	6 (2.61)	1 (1.56)
Beta-blocker										
N (%)	0 (0)	0 (0)	1 (1.06)	0 (0)	0 (0)	0 (0)	0 (0)	5 (3.70)	5 (2.17)	0 (0)
None										
N (%)	64 (98.46)	29 (100.00)	84 (89.36)	34 (94.44)	21 (95.45)	9 (90.00)	86 (98.85)	125 (92.59)	215 (93.48)	62 (96.88)

ALS (Amyotrophic lateral sclerosis), other MNDs (other motor neuron diseases), PD (Parkinson’s disease), other NDDs (other neurodegenerative diseases), NMDs (neuromuscular diseases), Tremor, Inflamm. CNS dis. (inflammatory/ autoimmune/ infectious central nervous system diseases), VCDs (vascular central nervous system diseases), other NDs (other neurological diseases) and Control group (non-neurological control group). *NA* not applicable

^A^multiple entries possible

### Tear fluid sampling

TF collection was performed following a standardized protocol. The standard operating procedure for the tear fluid collection (S2) as well as a video (S3) explaining the process can be found in the supplementary material. In brief, collection was performed using STT strips (Madhu Instruments Pvt. Ltd., New Dehli, India), with 41 mm length and 5 mm width. Non-sterile gloves were worn during the entire sampling procedure. The 5 mm long, proximal, rounded part of the strip was placed in the lower fornix of each eye near the lateral canthus, distant to the cornea, and left in place with eyes closed. After 10 min, the strips were carefully removed and the WL in mm for both eyes was noted. The strips were each individually packed tightly in 0.75 ml sample storage tubes (Micronic, Lelystad, Netherlands) and immediately stored at -20 °C and transferred to -80 °C within one week for further analysis. Measurements were performed between 9 am and 5 pm and at a room temperature between 20–25 °C. Previous history regarding eye diseases, eye medications, the use of contact lenses and the sensation of dry, irritated or painful eyes was recorded on a standardized documentation sheet and the information was entered into a digital database.

### Statistical analysis

Statistical analyses were performed using R version 4.0.4 (The R Foundation for statistical Computing, Vienna, Austria) and RStudio Version 1.4.1717S (Posit Software, PBC, Boston, MA). The significance level was set at α = 0.05 (5%). For the overall cohort, categorical data are described by absolute and relative frequencies, and quantitative data by mean with standard deviation or median with minimum and maximum.

The mean value of the WL in mm/10 min between both eyes was calculated for each subject. To distinguish the mean values of the WL between different groups, multiple linear regression was performed, taking into account the confounders age and sex as well as specific eye diseases, eye medications and systemic medications when mentioned as independent variables. Kruskal–Wallis tests were performed to compare distributions of relevant variables between groups (age groups, eye diseases, dry eye sensation). Subsequent post-hoc tests for these pairwise group comparisons were performed using the Bonferroni method to avoid alpha accumulation caused by multiple testing. Pearson's correlation coefficient was used to estimate the association between clinical and environmental data and TF production unless for ordinal parameters where Kendall’s correlation coefficient was used. To conduct these correlation analyses, we relied on local daily climate data from “Deutscher Wetterdienst” [34]. For relevant effect measures, 95% confidence intervals were calculated.

## Results

**Fig. 1:**
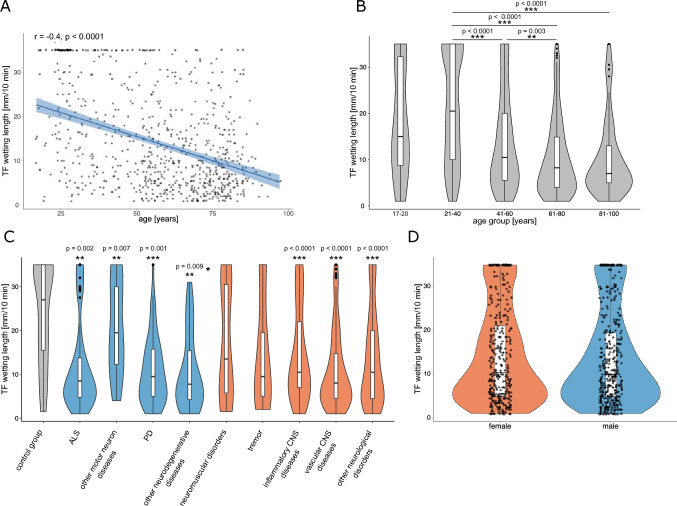
Analysis of TF WL in different ND groups. **A** Using Pearson’s correlation, a moderate negative correlation between WL (mm/10 min) and age (years) was observed (n = 772). Regression line is shown in blue with 95% confidence intervals in light blue as well as all individual data points. **B** Analysis of the distribution of TF WL in mm/10 min in five different age groups revealed significant differences between the age groups 21–40 (n = 167) and 41–60 (n = 208), 21–40 and 61–80 (n = 302), 21–40 and 81–100 (n = 80) and between age groups 41–60 and 61–80 (Kruskal–Wallis test and pairwise Wilcoxon rank sum test with Bonferroni post-hoc testing). Data is displayed in violin plots with inlayed box plots. **C** Division of the total cohort into 9 disease subgroups (NDDs are shown in blue, other NDs in red) and a control group without signs of ND (grey). Comparison of mean WL in the 9 subgroups with the control group, using age- and sex-corrected multiple linear regression analysis, revealed significantly reduced TF production in 7 of 9 groups compared to controls: ALS, other MNDs, PD, other NDDs, inflammatory/ autoimmune/ infectious CNS diseases, VCDs and other NDs. Data is displayed in violin plots with inlayed box plots. **D** Comparison of wetting lengths in female (red) and male (blue) subjects showed no significant difference using age-corrected multiple linear regression analysis. Data is displayed in violin plots with inlayed box plots as well as all individual data points. Inlayed boxplots for all graphs show median and 1^st^ and 3.^rd^ quartile. Observations with a distance from the border of the box larger than 1.5 × interquartile range are shown as individual dots; *p < 0.05, **p < 0.01, ***p < 0.001

TF was collected from a total of 772 subjects, of which 708 were patients with NDs and 64 were healthy controls. Detailed demographic characteristics and medical history regarding ocular pathologies is given in supplementary Table S1. We first assessed age distribution in the overall cohort and the effect of age on TF production. A correlation analysis between age and WL in mm/10 min in the overall cohort showed a moderate negative correlation (r_P_ =  – 0.40, p < 0.0001, 95% CI [ –0.46, – 0.34]) (Fig. 1A), while sex had no significant effect on TF production (males: mean value ± sd 14.19 mm/10 min ± 10.74, n = 344; females: 13.45 mm/10 min ± 10.54, n = 407) (p = 0.4, 95% Mann–Whitney-U test) (Fig. 1D). Significant differences were identified using pairwise Wilcoxon rank sum test adjusting for multiple testing using the Bonferroni method between the age groups 21–40 years (21.0 ± 11.6 mm/10 min, n = 167) and 41–60 years (13.8 ± 10.3 mm/10 min, n = 208) (p < 0.0001), 21–40 years and 61–80 years (10.5 ± 8.6 mm/10 min, n = 302) (p < 0.0001), 21–40 years and 81–100 years (10.3 ± 8.5 mm/10 min, n = 80) (p < 0.0001), and between the age groups 41–60 years and 61–80 years (p = 0.003) (Fig. 1B). We then compared the WL of patients in different disease groups as well as control subjects, adjusting for age and sex (Fig. 1C). Compared to the control group with no signs of NDs (23.8 ± 11.4 mm/10 min) there was a significant reduction in TF production in 7 out of 9 NDs groups: ALS (11.7 ± 10.4 mm/10 min, p = 0.002, estimate =  – 6.72, (95% CI [ – 10.84, – 2.60])), other MNDs (19.7 ± 10.3 mm/10 min, p = 0.007, estimate =  – 6.20, [ –10.63, – 1.77]), PD (10.8 ± 7.8 mm/10 min, p = 0.001, estimate =  – 5.90, [ –9.19, – 2.60]), other NDDs (10.7 ± 8.3 mm/10 min, p = 0.009, estimate =  – 5.87, [ – 10.21, – 1.53]), inflammatory/autoimmune/infectious CNS diseases (14.6 ± 10.6 mm/10 min, p < 0.0001, estimate =  – 9.98 [ – 12.99, – 6.97]), VCDs (10.8 ± 8.9 mm/10 min, p < 0.0001, estimate =  – 6.86 [ – 10.07, – 3.65]) and other NDs (13.5 ± 10.5 mm/10 min, p < 0.0001, estimate =  – 8.15, [ – 10.96, – 5.35]). No significant differences were observed for the groups of neuromuscular diseases and tremor syndromes. We also performed this analysis correcting for administration of prostaglandin agonist and beta blocker eye drops, which did not change the significances (S5).

We then investigated the influence of eye-related factors (contact lens use, dry eye sensation, or pre-existing ocular diseases) on TF production. A significant difference in TF production was observed between subjects with a cataract (n = 72, 9.0 ± 7.3 mm/10 min) and those without ocular disease (n = 595, 14.4 ± 10.9 mm/10 min) (p = 0.0004, pairwise Wilcoxon rank sum test). When correcting for age and sex in a multiple linear regression model, however, this effect did not reach significance. We further investigated the effect of ocular comorbidities in the subgroups mentioned in Table 1. In detail, we added the occurrence of eye diseases, namely cataract, glaucoma, inflammatory eye diseases (conjunctivitis, keratitis, uveitis) and Sjögren’s syndrome in the different disease groups to the above mentioned multiple linear regression model in a binary manner. The significance of the group variable persisted in these models (S5).

**Fig. 2:**
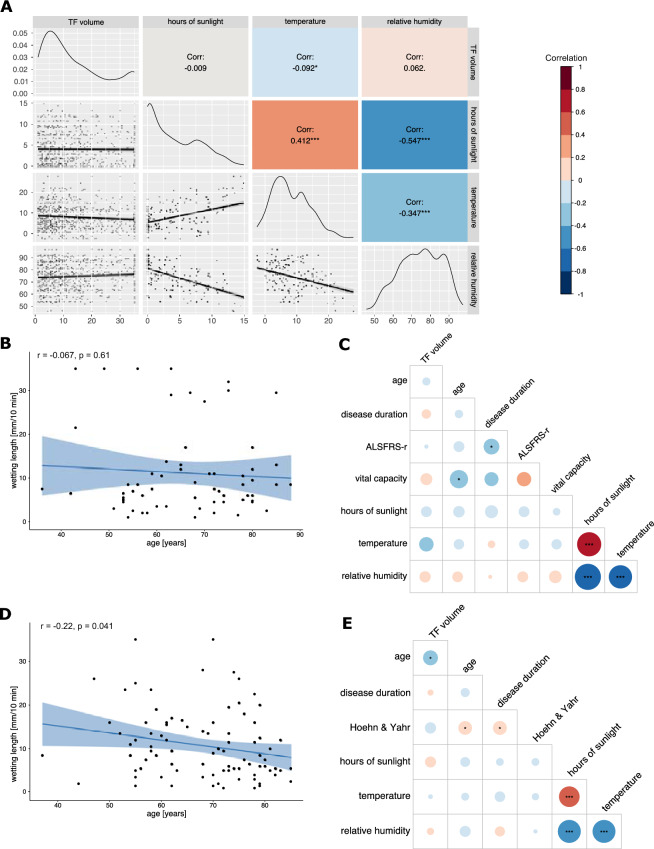

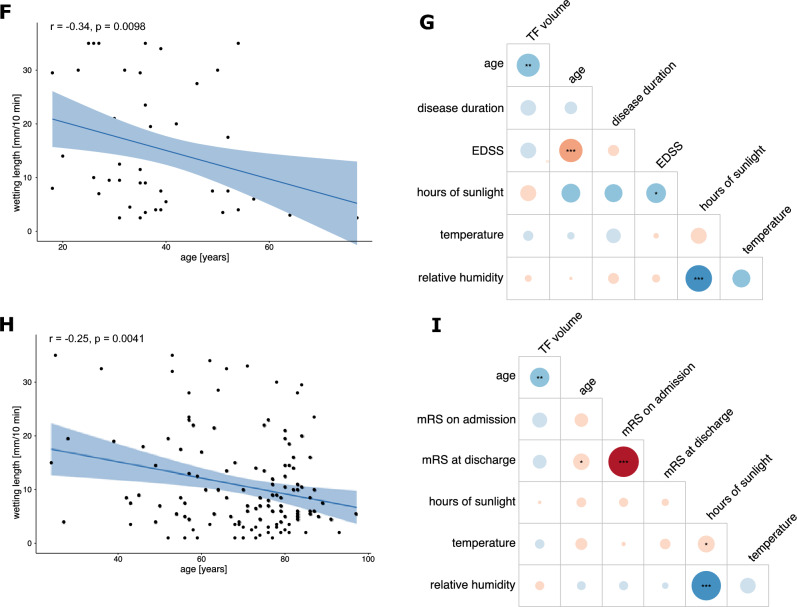
Correlation analysis between TF WL, environmental and clinical parameters in the overall cohort and the PD, ALS, MS and VCDs subgroups **A** Correlogram depicting the Pearson’s Correlation between TF volume and hours of sunlight, temperature and relative humidity in the overall cohort (n = 772). A weak, significant correlation between temperature and TF volume is shown. Upper half notes the correlation and the corresponding significance levels indicated by asterisks, the diagonal row shows the data distribution and the bottom half shows the regression line with 95% confidence intervals and individual data points. **B**, **D**, **F**, **H** Pearson correlation between TF volume and age in the ALS (**B**), PD (**D**), MS (**F**) and VCDs (**H**) subgroups. Regression line is shown in blue with 95% confidence interval in light blue as well as all individual data points. **C**, **E**, **G**, **I** Correlation plots between TF WL, clinical and meteorological data for the ALS (C, n = 65, PD (E, n = 94), MS (G, n = 56) and VCDs (I, n = 135) cohort respectively. Size and color of the circles correlate to the strength of the correlation. Significances are shown as asterisks inside the circles. Pearson’s correlation coefficient is shown for all but Hoehn & Yahr scale, EDSS and mRS, for which Kendall’s tau is displayed. Significance levels are shown as *p < 0.05, **p < 0.01, ***p < 0.001 for all. The correlation index color legend is applicable for all correlograms

Further, we evaluated the correlation between WL and environmental factors, such as time of collection and meteorological data. There was a weak significant correlation between WL and temperature (r_P_ =  – 0.09, p = 0.01, 95% CI [ – 0.16, – 0.02]), but none between hours of sunshine or relative air humidity and WL (Fig. 2A).

Patients with NDDs represented a particularly large subpopulation in our cohort (n = 224 including patients with ALS and other MNDs, PD, and other NDDs such as dementia, choreatic syndrome, spinocerebellar ataxia), which prompted us to perform a subgroup analysis in this particular patient group. WL in the NDDs group was 12.2 ± 9.4 mm/10 min and in the control group with no signs of NDDs 23.8 ± 11.4 mm/10 min (n = 64) (p < 0.0001, estimate =  – 7.41 (95% CI [ – 10.26, – 4.56])), multiple linear regression, age- and sex-adjusted).

Because PD and ALS are exemplary NDDs with chronic and rapid progression, we performed a more in-detail analysis of this patient group (clinical data in Table 2). In both subgroups, WL was significantly reduced compared to the healthy controls considering age- and sex-adjusted multiple linear regression.

**Table 2:** Demographic data and clinical characteristics of the subgroups studied in detail

	ALS	PD	MS	VCD	Control group
N	65	94	56	135	64
Sex					
Male					
N (%)	38 (58.46)	58 (61.70)	18 (32.14)	51 (37.78)	26 (40.63)
Female					
N (%)	27 (41.54)	36 (38.30)	38 (67.86)	84 (62.22)	38 (59.38)
Age					
Mean ± sd	65.26 ± 12.72	68.10 ± 10.53	40.36 ± 13.52	69.96 ± 14.80	45.30 ± 21.06
Median (min–max)	66 (36–88)	70 (37–85)	37.50 (18–77)	74 (24–97)	36 (22–88)
Clinical data					
Disease duration (y)					
Mean ± sd	2.74 ± 4.54	6.48 ± 5.63	5.91 ± 8.92	0.42 ± 1.73	NA
Median (min–max)	1.58 (0.25–33.58)	5.08 (0–25)	2.71 (0–49.83)	0 (0–14.50)	NA
Stratum of onset					
Spinal					
N (%)	42 (65.62)	NA	NA	NA	NA
Bulbar					
N (%)	18 (27.69)	NA	NA	NA	NA
Simult. spinal/bulbar					
N (%)	1 (1.54)	NA	NA	NA	NA
Axial/respiratory					
N (%)	2 (3.08)	NA	NA	NA	NA
FTD					
N (%)	1 (1.54)	NA	NA	NA	NA
Unknown					
N (%)	1 (1.54)	NA	NA	NA	NA
ALSFRS-R score					
Mean ± sd	36.40 ± 7.61	NA	NA	NA	NA
Median (min–max)	38 (12–48)	NA	NA	NA	NA
Vital capacity [ml]					
Mean ± sd	2646.43 ± 1196.93	NA	NA	NA	NA
Median (min–max)	2500 (500–4700)	NA	NA	NA	NA
Hoehn and Yahr stage					
Mean ± sd	NA	2.32 ± 0.97	NA	NA	NA
Median (min–max)	NA	2 (1–5)	NA	NA	NA
EDSS					
Mean ± sd	NA	NA	2.87 ± 1.73	NA	NA
Median (min–max)	NA	NA	2.5 (1–7.5)	NA	NA
mRS at time of admission					
Mean ± sd	NA	NA	NA	2.41 ± 1.72	NA
Median (min–max)	NA	NA	NA	2.5 (0–5)	NA
mRS at time of discharge					
Mean ± sd	NA	NA	NA	1.66 ± 1.42	NA
Median (min–max)	NA	NA	NA	1 (0–5)	NA
Ophthalmological data					
Wetting length [mm/10 min]					
Mean ± sd	11.70 ± 10.42	10.82 ± 7.78	14.96 ± 10.50	10.84 ± 8.85	23.79 ± 11.42
Median (min–max)	8 (1–35)	9.50 (1–35)	11.25 (2.5–35)	8 (1–35)	27 (1.5- 35)
Feeling of dry eyes					
Strong					
N (%)	0 (0)	4 (4.26)	1 (1.79)	7 (5.19)	4 (6.25)
Medium					
N (%)	9 (13.85)	26 (27.66)	10 (17.86)	28 (20.74)	10 (15.63)
Low					
N (%)	4 (6.15)	13 (13.83)	10 (17.86)	19 (14.07)	16 (25.00)
None					
N (%)	33 (50.77)	43 (45.74)	30 (53.57)	69 (51.11)	34 (53.13)
Unknown					
N (%)	19 (29.23)	8 (8.51)	5 (8.93)	12 (8.89)	0 (0)
Ophthalmic disease					
Cataract					
N (%)	4 (6.15)	21 (22.34)	0 (0)	32 (23.70)	5 (7.81)
Glaucoma					
N (%)	3 (4.62)	6 (6.38)	0 (0)	7 (5.19)	0 (0)
None					
N (%)	57 (87.69)	65 (69.15)	49 (87.50)	90 (66.67)	58 (90.63)

The ALS (Amyotrophic lateral sclerosis), PD (Parkinson’s disease), MS (Multiple sclerosis), VCDs (vascular central nervous system diseases) subgroups and the Control cohort. *ALSFRS-r* Revised Amyotrophic Lateral Sclerosis Functional Rating Scale; *EDSS* Expanded Disability Status Scale; *mRS* Modified Rankin Score; *NA* not applicable

In the subgroup of patients with ALS, the comparison of WL between bulbar-onset (n = 18, 11.7 ± 10.5 mm/10 min) and spinal-onset (n = 42, 11.6 ± 10.4 mm/10 min) patients showed no significant difference (p = 0.6, estimate =  – 1.59 (95% CI [ – 7.53, 4.36]), age and sex adjusted multiple linear regression). In contrast to the effects seen in the overall cohort, WL did not correlate significantly with age in this subgroup (Fig. 2B). WL also did not significantly correlate with disease duration or disease severity (quantified by the revised ALS-Functional Rating Scale (ALSFRS-r)) nor with environmental data, although a trend for a weak negative correlation of temperature and WL was observed (Fig. 2C). Correlation analyses in the PD cohort displayed a weak negative correlation of age and WL (r_P_ =  – 0.22, p = 0.04, 95% CI [ – 0.41, – 0.01]), similar to the moderate correlation observed in the entire cohort (Fig. 2D). WL did not significantly correlate with disease duration, Hoehn & Yahr scale or environmental data (Fig. 2E).

We also performed subgroup analyses in two further large disease groups, namely patients with MS and VCDs (clinical data in Table 2).

WL in the subgroup of patients with MS (n = 56, 15.0 ± 10.5 mm/10 min) was significantly reduced compared to the control group (23.8 ± 11.4 mm/10 min) using age- and sex-adjusted multiple linear regression (p < 0.0001, estimate =  – 10.38 (95% CI [ – 13.81, – 6.94])) (Fig. 2F). Correlation analyses in this subgroup of patients showed a negative correlation between TF volume and age as observed in the entire cohort (r_P_ =  – 0.3, p = 0.01, 95% CI [ – 0.5, – 0.09]) as well as a negative trend between TF volume and disease duration (r_P_ =  – 0.2, p = 0.1, 95% CI[ – 0.4, 0.1]) and Expanded Disability Status Scale (EDSS) (tau =  – 0.2, p = 0.1) (Fig. 2G).

135 patients with VCDs were also included in the analysis. WL of patients with VCDs was significantly reduced compared to the control group using age and sex corrected multiple linear regression. A negative correlation between TF volume and age (r_P_ =  – 0.2, p = 0.004, 95% CI [ – 0.4, – 0.08]) as well as modified Rankin Scale (mRS) scores on admission (tau =  – 0.1, p = 0.05) and at discharge (tau =  – 0.1, p = 0.1) was observed (Fig. 2H, I).

Lastly, we investigated whether the effects on TF wetting length in these four subgroups were influenced by systemic medication taken by the patients. We therefore evaluated the influence of typical systemic treatment options in the subgroups. An overview over the medications evaluated in detail can be found in Table S4. Most medications showed no significant influence on the TF volume in the observed subgroups when also correcting for age and sex. Interestingly, when correcting for age and sex as well as the medications taken using a multiple linear regression, the administration of monoamine oxidase type B (MAO-B) inhibitors was associated with significantly higher wetting lengths in the PD group (17.5 ± 10.6 mm/10 min for MAO-B inhibitor taking patients, 9.9 ± 7.2 mm/10 min for non-MAO-B inhibitor taking patients, p = 0.001, estimate = 8.69 [3.61, 13.77]). Similarly, the administration of systemic corticosteroids was associated with significantly higher wetting lengths in the group of patients with MS (17.2 ± 10.6 mm/10 min for corticosteroid receiving patients, 9.4 ± 8.2 mm/10 min for non-corticosteroid receiving patients, p = 0.044, estimate = 6.84 [0.31, 13.29]).

## Discussion

Several recent studies have demonstrated the potential of TF as a biomarker source in NDs, but comprehensive information on TF production in this heterogeneous patient group so far was not available. To our best knowledge, our cross-sectional study provides data on the largest TF collection to date in a well-phenotyped cohort of 708 patients with NDs. To avoid bias introduced during collection, we used a highly standardized protocol to harmonize collection conditions. In addition, clinical severity scores that were acquired for patients with ALS, PD, MS and VCDs allowed us to perform more detailed correlation analyses in these subgroups.

Overall, we observed a strong negative correlation of WL and age, which is in line with previously published studies reporting a decrease of TF production with age [30, 32, 34, 39]. Our results thus confirm age as a confounder for WL in neurological patients, which needs to be considered in future studies.

Next, we assessed the influence of environmental factors on TF production. We did not observe any significant correlation between air humidity and TF production. In previous studies, reduced air humidity has been shown to affect the TF volume via increasing evaporation [1, 9, 40]. Our information on humidity relied on local outdoor daily climate data from the national German weather service [15] and was not measured in an environmental chamber. Our assumptions about the humidity at time of sampling might therefore differ somewhat from the actual condition indoors. Previously published data on humidity and duration of exposure needed to affect TF volume are inconclusive [1, 9]. There was a significant negative correlation between TF production and temperature, which has been described in previous studies and may be due to increased evaporation of TF at warm temperatures [2, 40]. Taken together, our data suggest that in our cohort the effect of environmental factors on TF production was relatively small.

We then compared the WL between different disease groups to a control group with no evidence of NDs. Subdividing the overall cohort into patients with and without NDD, we observed significantly reduced WL in patients with NDDs, even when correcting for age and sex. Significantly reduced WL were observed in patients with PD, ALS, other NDDs (including atypical PS, dementias and chorea), VCDs, inflammatory/ autoimmune/ infectious CNS diseases and other NDs compared to the control group when corrected for age and sex.

Our report on reduced WL in patients with VCDs is in line with previous studies of patients with ischemic stroke [25, 39]. Data on TF volume in patients with inflammatory CNS diseases is sparse, but to the best of our knowledge we show here, for the first time, reduced WL in the subgroup of patients with inflammatory/ autoimmune/ infectious CNS diseases and a similar trend in the subgroup of patients with MS.

We also evaluated the effect of topical medication, namely prostaglandin agonist and beta blocker eye drops on the TF volume by including them as coefficients in our multiple linear regression model. These drugs used for the treatment of glaucoma have been shown to decrease TF volume in large cohort of patients [19, 41]. In our cohort, the significant difference in TF volume between the subgroups mentioned in Table 1 and the control group persisted and none of the medications showed a significant contribution to the model. Similarly, we analyzed the effect of eye diseases, namely cataract, glaucoma, inflammatory eye diseases (conjunctivitis, keratitis, uveitis) and Sjögren’s syndrome on the TF volume in these subgroups. None of the mentioned diseases entities had a significant contribution to the multiple linear regression models, which continued to show a significant effect of the group (control group or disease group) on the TF volume, even when taking the ocular diseases into account. Some ocular diseases are associated with dry eye symptoms themselves, such as allergic conjunctivitis or Sjögren’s syndrome [3, 5]. However, the number of patients with eye diseases in our cohort was relatively small, possibly explaining our results.

Of particular interest to us were the subgroups comprising patients with NDDs. For many of these disorders the diagnosis is still challenging and mainly based on the evaluation of clinical symptoms [10, 35], resulting in initial misdiagnoses and long diagnostic timelines [33, 35]. In light of current developments for effective disease-modifying treatments, there is a great need for biomarkers that can facilitate early diagnosis and early therapeutic intervention [36]. Tear fluid could represent an auspicious substrate here [17, 29].

For many NDDs, it has been postulated that the pathology spreads through different regions of the brain, including the brainstem, as for example in PD and ALS [7, 8]. Of particular interest in regard to the regulation of TF production is the hypothalamus, as an important control unit of the autonomic nervous system, and the brainstem, with autonomic core areas such as the superior salivatory nucleus. It is therefore tempting to speculate that the neurodegenerative process in these disorders influences TF production. Both ALS and PD show pathological abnormalities in these anatomical areas. In ALS, atrophy of the hypothalamus, even in pre-symptomatic gene mutation carriers [16], TAR DNA binding protein (TDP-43) pathology in various hypothalamic areas [13] and in core areas of the brainstem even in early stages of the disease [8] has been demonstrated. Patients with PD show pathological accumulation of alpha-synuclein in the hypothalamus and the brainstem [7, 26]. Beginning in the olfactory bulb and the lower brainstem in the pre-symptomatic phase, the pathology spreads cranially [7]. This leads to non-motor symptoms such as autonomic dysfunction and sleep disturbances, often many years before the first motor symptoms appear [37].

Here we demonstrate reduced WL in patients with PD. Several studies report WL using a non-anaesthetized version of the STT in cohorts of varying size with differing results ranging from unchanged to decreased WL [11, 27, 29]. Interestingly, Hamm-Alvarez et al. reported two different cohorts—one studying reflex tears, in which the WL were significantly lower in patients with PD compared to controls [17], and one evaluating basal tears, in which no significant difference in WL could be observed [18]. Basal tears are collected using local anesthetics on the ocular surface to prevent reflective secretion, whereas for reflex tears no anesthetics are used, increasing stimulation of TF secretion via a parasympathetic feedback loop involving regions in the brain stem. Dysregulation of parasympathetic innervation of the lacrimal gland potentially shown by effects on reflective but not basal tear production could thus be a potential sign of PD-associated pathology in the brain stem.

We also report significantly reduced TF volume in ALS patients and patients with other MNDs compared to the control group. The significant reduction of TF production in both ALS and PD patients suggests that this may represent another non-motor sign. Interestingly, the reduced WL occurs independently of disease duration and severity, possibly indicating that corresponding areas responsible for TF production are already affected at an early stage of disease. WL did not significantly differ between patients with bulbar or spinal onset of symptoms, although this might be an effect of small sample size (n = 18 and 42, respectively). Further studies are needed to provide more insights in these subgroups.

Importantly, except for a trend toward a weak negative correlation between temperature and WL in the ALS group, that was also observed in the overall cohort, no further correlations were detected between environmental factors and the four disease groups that were studied in more detail: ALS, PD, VCDs and MS. Considering that the measurements took place within the hospital building and especially since some inpatients had already been in the hospital for several days, less influence of external environmental changes is likely.

Importantly, none of the standard treatment options for these diseases had a significant effect on the reduction of TF volume. This is contrary to previous reports [19, 41]. While drugs for the treatment of peptic ulcer and gastro-esophageal reflux disease and anticholinergic drugs showed the most pronounced effect on the decrease of TF volume, smaller effects were also seen for antithrombotic agents, most forms of antihypertensive drugs, systemic corticosteroids as well as immunosuppressants. Interestingly, in our cohort, systemic corticosteroids in the MS group were associated with higher TF volume, which might be due to the relatively small sample size. To our knowledge this is also the first study reporting the intake of MAO-B inhibitors to be associated with higher TF volume, though this as well might be due to the age structure and relatively small sample size in this cohort. Further detailed studies of the different subgroups are needed to clarify the effects of systemic treatment options on TF volume.

There are several limitations to this study, including a possible selection bias as patients with dry eyes or other ophthalmologic comorbidities might be less inclined to participate voluntarily to avoid discomfort by the STT. Another bias is the monocentric, cross-sectional design of the study. Further studies with multicentric sample acquisition and prospective long-term follow-up of selected disease groups will be needed to generate a more comprehensive picture on the robustness of the described effects.

In summary, this study demonstrates alterations in WL in neurodegenerative, neurovascular and neuroinflammatory diseases, mostly independent from environmental factors and evaluated systemic and topical treatment options as well as ocular diseases. Furthermore, we showed that TF sampling can be performed reproducibly on a large clinical cohort, opening the possibility of studying the collected TF samples for biomarker molecules in NDs.

### Supplementary Information

Below is the link to the electronic supplementary material.

Supplementary file1 (XLSX 16 KB)

Supplementary file2 (MP4 63107 KB)

Supplementary file3 (DOCX 622 KB)

Supplementary file4 (XLSX 11 KB)

## Data Availability

Aggregated data is available from the authors upon reasonable request.
