# Supplementary material for: Reduced tear fluid production in neurological diseases: a cohort study in 708 patients
Source: J Neurol. 2023 Dec 8;271(4):1824–36. doi: 10.1007/s00415-023-12104-3 (PMC10973005; doi:10.1007/s00415-023-12104-3)

**Supplementary Material**

**Reduced tear fluid production in neurological diseases – A cohort study in 708 patients**

Elena Luib*^1^, Antonia F. Demleitner*^1^, Isabell Cordts^1^, Erica Westenberg^1^, Petra Rau^1^, Dominik Pürner^1^, Bernhard Haller^2^, Paul Lingor^1,3,4,†^

*These authors contributed equally

^1^ Department of Neurology, Klinikum rechts der Isar, School of Medicine, Technical University of Munich, Munich, Germany

^2^ Institute of AI and Informatics in Medicine, Klinikum rechts der Isar, Technical University of Munich, Munich, Germany

^3^ DZNE, German Center for Neurodegenerative Diseases, Munich, Germany

^4^ Munich Cluster for Systems Neurology (SyNergy), Munich, Germany

^†^Corresponding author: Paul Lingor, paul.lingor@tum.de, Department of Neurology, Klinikum rechts der Isar, School of Medicine, Technical University of Munich, Ismaninger Str. 22, 81675 München

Table of Contents

[Supplementary 1. Table of demographic and clinical data of the disease groups and subgroups 3](#_Toc148339141)

[Supplementary 2. Standard Operating Procedure (SOP) used for tear fluid collection of the study 4](#_Toc148339142)

[Supplementary 3. Descriptive video showing requirements and procedure of tear fluid collections used for the study 7](#_Toc148339143)

[Supplementary 4. Table of systemic, topical medications and eye diseases in the four subgroups studied in detail 7](#_Toc148339144)

[Supplementary 5. Comparison of the different multiple linear regression models 7](#_Toc148339145)

# Supplementary 1. Table of demographic and clinical data of the disease groups and subgroups

Demographic and clinical data of the disease groups in bold and subgroups: MNDs (motor neuron disease), ALS (Amyotrophic lateral sclerosis), HSP (hereditary spastic paraplegia), SMA (spinal muscular atrophy), SBMA (spinobulbar muscular atrophy), possible MNDs (possible motor neuron disease), NMDs (neuromuscular diseases), Myasthenia gravis, Myopathy, other NMDs (other neuromuscular diseases), MDs (movement disorders), PD (Parkinson’s disease), aPD (atypical and secondary Parkinson’s disease), gait disorder, tremor syndrome, other MD (other movement disorders), Dementia, Inflamm. CNS dis. (inflammatory/ autoimmune/ infectious central nervous system diseases), multiple sclerosis, infectious CNS diseases, other inflammatory CNS diseases, VCDs (vascular central nervous system diseases), TIA/Stroke (transient ischaemic attack or stroke), intracranial haemorrhage, sinus vein thrombosis, other vascular CNS diseases, epilepsy, malignoma, head and facial pain, vertigo syndromes, diseases of the PNS (peripheral nervous system), other NDs (other neurological diseases) and Control group (non-neurological control group). NA, not applicable.

# Supplementary 2. Standard Operating Procedure (SOP) used for tear fluid collection of the study

**Standard Operating Procedure (SOP)**

**Collection and processing of tear fluid**


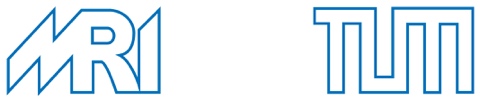

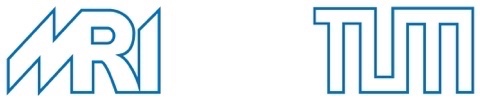


Technische Universität München

Klinikum rechts der Isar

Version 1.0, December 12^th^ 2019

1. **Consent from test subject for acquisition of tear fluid for the biobank**

- Obtain written consent from test subject using the current biobank informed consent form (*ICF v4.0 as of 02/15/2019*)
- Hand copy of ICF to test subject

1. **Collection of tear fluid (TF)**
   1. **Material preparation**

**Necessary Material**

| **Product** | **Product specifications** |
| --- | --- |
| Sterile Schirmer test strips without dye (STS) | Schirmer Tear Test Ophthalmic Strips, OptiTech Eyecare, Tarun Enterprises |
| Collection tubes with serial number and pink and blue lid |  |
| Non-sterile gloves |  |
| Ice box |  |
| Timer |  |
| Sample storage bags |  |
| Data collection form |  |
| ICF |  |
| Sticker with case ID |  |

**Documentation**

- Sticker with the case ID is affixed to the data collection form
- Data collection form is filled with the sample collection details
- Date and time of sampling
- Availability of consent form
- Serial number of sample collection tubes (pink lid for left eye, blue lid for right eye)

**Material**

- The STS is bent inside the packaging at the start of the scale
  1. **Sampling**
- Hands are disinfected and non-sterile gloves put on
- STS are carefully removed from packaging
- Starting at the left eye, the lower eyelid is gently pulled down and the subject asked to look up. The bent, rounded end of the strip is inserted into the lower eyelid mediolaterally (2-3 mm from lateral canthus of the eye)
  - *Do not touch the cornea to prevent artificial stimulation of tear production*
  - *Avoid skin contact of the lower end of the strip to prevent contamination*
- Procedure is repeated within 20 s for the right eye and subject asked to close their eyes
- STS are left in eyes for 10 min or until strips are full
  - *Note premature filling on data collection form*
- STS is carefully removed beginning with the left eye and wetting length (in mm) is noted on data collection form
- Test strips are folded in half and placed in the designated sample collection tubes, the lids closed and the tubes placed on ice
- Clinical information is noted on the data collection form
- Fasting status
- Diagnosis
- Known local or systemic eye diseases or eye surgeries
- Administered ocular medication within the last 12 h
- Wearing of contact lenses within the last 12 h
- Subjective perception of dry eyes, irritated or painful eyes on a categorical scale of 0-3 (0 = no discomfort, 1 = mild discomfort, 2 = moderate discomfort, 3 = severe discomfort)
  1. **Sample storage**
- Data collection form, ICF and sample collection tubes are placed in one sample storage bag
- Sample storage bags are transferred to a -20°C freezer
- Mark freezing time on sample storage bag
  - *Time between sample collection and freezing should not exceed one hour*

1. **Further processing of the samples**
   1. **Registration of patient and sample in the biobank database**

- Enter available data on data collection form into biobank database and generate/assign kit ID to samples
- Place samples in the current rack and note rack position and kit ID on data collection form
- The data collection and informed consent from is filed in the corresponding folders
  1. **Scanning the rack**
- The rack is scanned by an automated QR/barcode scanner to register and assign sample and rack position and type
  1. **Preservation of samples**
- Once completely filled, the rack is transferred to the designated drawer and freezer position at -80°C and preserved until further use or shipment of the samples

# Supplementary 3. Descriptive video showing requirements and procedure of tear fluid collections used for the study

File name: Luib et al. 2023 TF Wetting length Supplementary Video

# Supplementary 4. Table of systemic, topical medications and eye diseases in the four subgroups studied in detail

ALS (Amyotrophic lateral sclerosis), PD (Parkinson’s disease), MS (Multiple sclerosis), VCDs (vascular central nervous system diseases) subgroups. For ophthalmic diseases, eye medication and systemic medication multiple entries are possible; NA, not applicable

# Supplementary 5. Comparison of the different multiple linear regression models

Next to each other, the three models are shown: to the left the original, only correcting for age, sex and the group (disease vs. control), in the middle one correcting for eye diseases and to the right one correcting for topical eye medication as well. Only calculable coefficients (e.g. with at least one positive entry) are shown. Screenshots were taken from the analysis using the tab_model() function from the package sjPlot (version 2.8.15) in R.

Amyotrophic lateral sclerosis vs. control


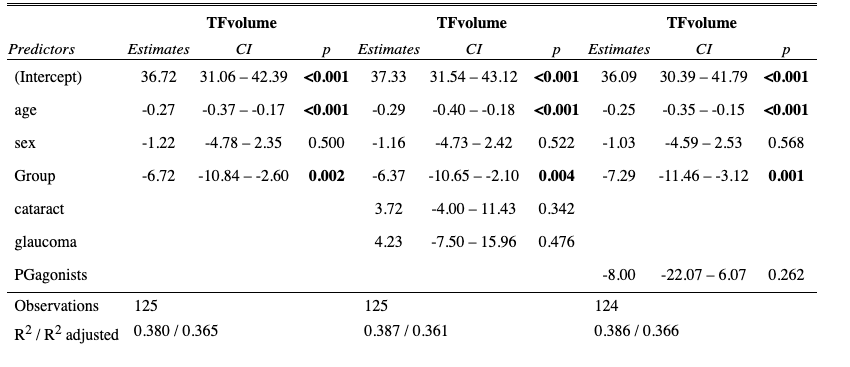


Other motor neuron diseases vs. control


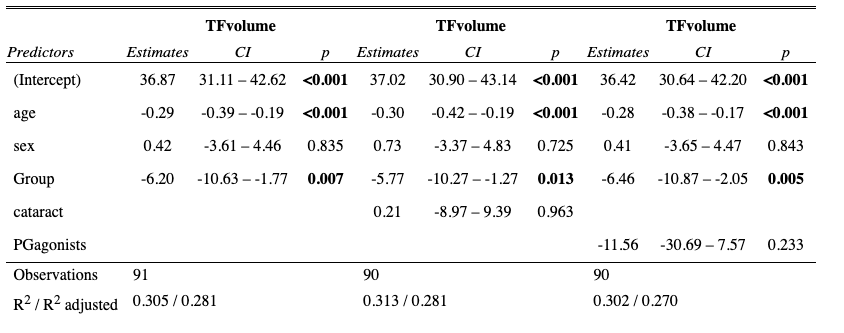


Parkinson’s disease vs. control


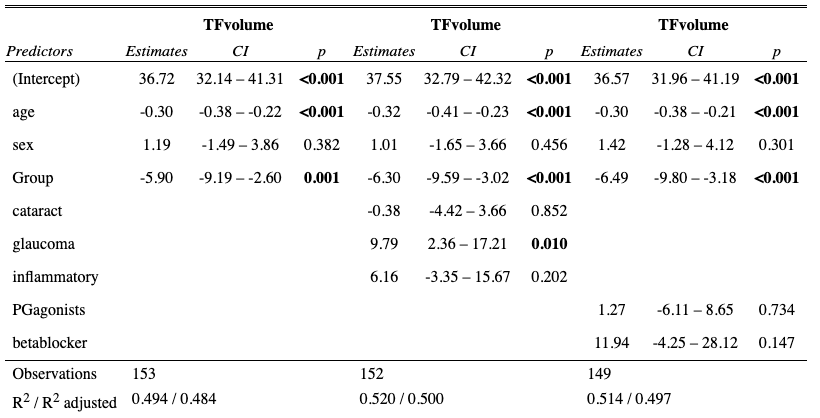


Other neurodegenerative diseases vs. control


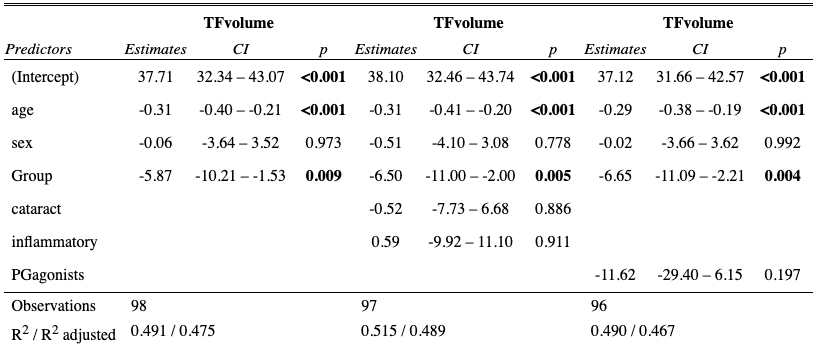


Neuromuscular diseases vs. control


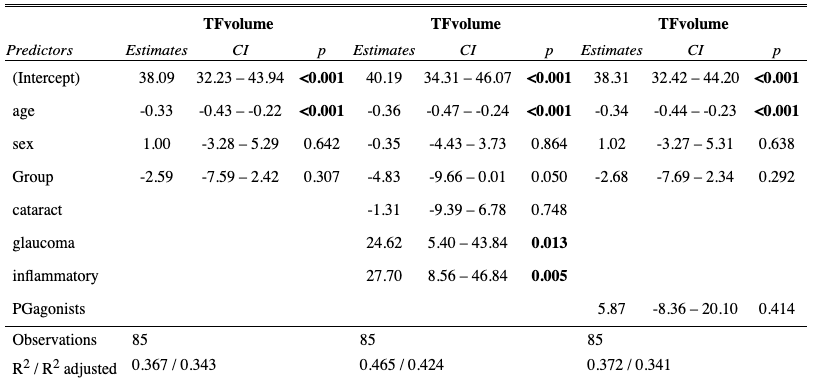


Tremor vs. control


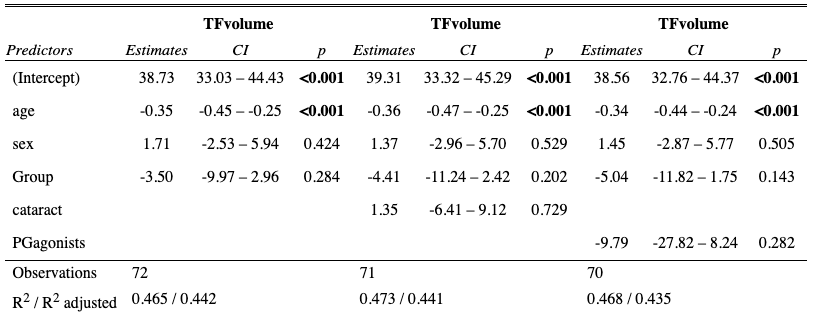


Inflammatory CNS diseases vs. control


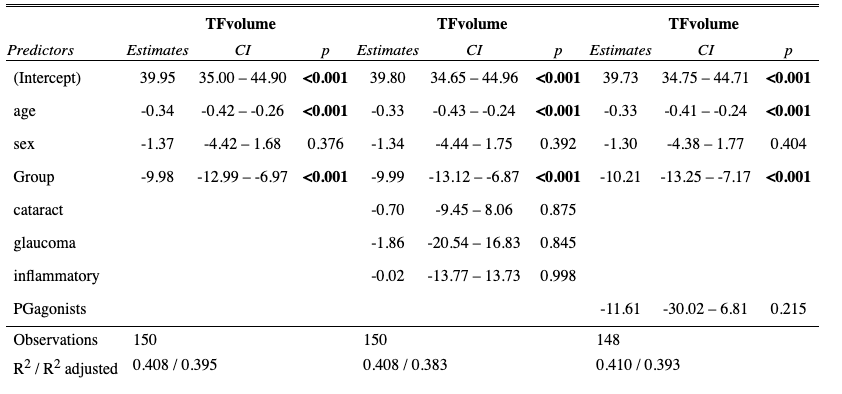


Vascular central nervous system diseases vs. control


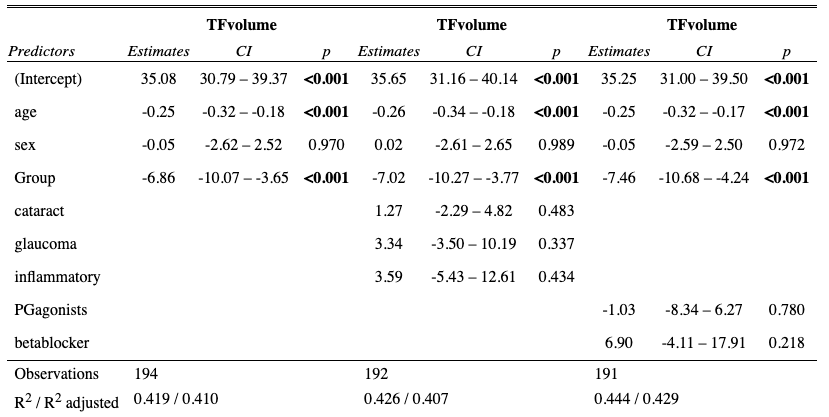


Other neurological diseases vs. control


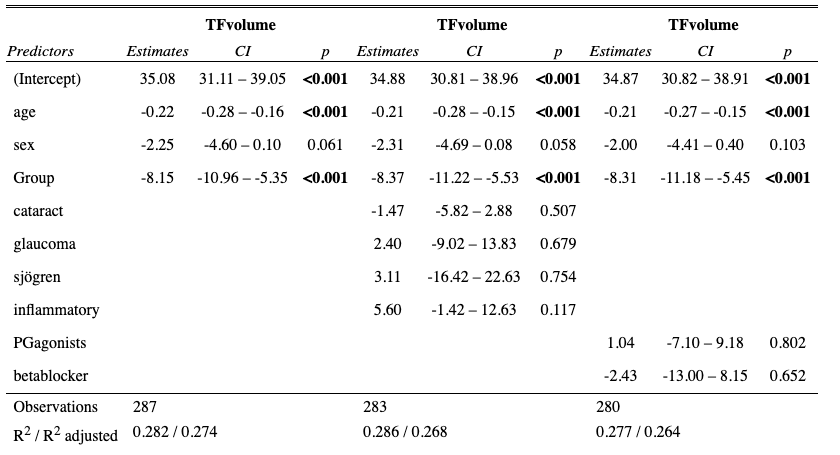

Supplement: Supplementary file 3 — Supplementary file3 (DOCX 622 KB) [file 415_2023_12104_MOESM3_ESM.docx]
